# Spectrophotometric Assay of Mefenamic Acid in Tablets Using 1,4-dioxane as Solvent for Extraction

**DOI:** 10.4103/0250-474X.73928

**Published:** 2010

**Authors:** G. Mathai, J. T. Moolayil, K. B. Jose, V. S. Sebastian

**Affiliations:** Department of Chemistry, Sacred Heart College, Thevara, Kochi- 682 013, India

**Keywords:** Dioxane, mefenamic acid, spectrophotometric assay

## Abstract

A spectrophotometric method of assay of mefenamic acid in tablets involving, dissolving the tablet powder in 1,4-dioxane and measuring the absorbance at 353.2 nm. The concentration of mefenamic acid is determined using the previously prepared calibration curve using standard solution of mefenamic acid in dioxane. The method was tested by assay of six different commercial tablets containing mefenamic acid.

Mefenamic acid, N-(2,3-xylyl)-2-aminobenzoic acid, is an analgesic used for the treatment of arthritic pain. Some of the spectrophotometric estimation methods include conversion to its colored iron complex[1]. The derivatization of aromatic ring by using coupling reaction with diazotized 4-amino-3,5-dinitrobenzoic acid has been performed prior to photometric estimation of mefenamic acid[2]. Spectrophotometric methods have been developed for the simultaneous estimation of mefenamic acid and paracetamol[3,4], mefenamic acid and ethamsylate[5,6] and mefenamic acid and drotaverine hydrochloride[7] in combination drugs. Mefenamic acid has a strong absorption band at 353 nm with high molar absorptivity but it has poor solubility in most organic solvents. We have observed that 1,4-dioxane is a good solvent for mefenamic acid but with low solubility of inactive ingredients in the tablet. Further, high boiling point (b.p. 100°) of dioxane provides accuracy for assay.

The solvent, 1,4-dioxane GR used for the experiment, was purchased from Merck Limited, Mumbai. Photometric experiments were performed by using Shimadzu double beam UV/Vis Spectrophotometer, model 1800, provided with quartz cells for sample and reference solution. The data were recorded with UV Probe software having the option for quantitative photometric measurements.

Mefenamic acid used as standard was obtained from Meftal 500 tablets by extraction with acetone. A standard solution of pure mefenamic acid was prepared by dissolving 0.0603g (0.25 mmol) of pure mefenamic acid in 100 ml of 1,4-dioxane. By suitable dilution five different standard solutions were made having concentrations of 0.25×10^-4^, 0.5×10^-4^, 1.25×10^-4^, 1.87×10^-4^and 2.5×10^-4^(mol/l). The absorbance of each solution was measured and a calibration curve was plotted using the UV Probe software.

Tablet powder equivalent to 0.0603 g (0.25 mmol) of mefenamic acid was accurately weighed out into 100 ml standard flask, 75 ml of 1,4-dioxane was added, shaken for 10 min and made up to 100 ml. The solution was allowed to stand for 10 min. A 1.0 ml aliquot of the solution was transferred into 20 ml standard flask and made up to the mark. The absorbance of 3 ml of the solution was measured at 353.2 nm, against a blank. The concentration of the solution was determined form the calibration curve. The results obtained are given in Table 1.

**TABLE 1:** ANALYSIS OF MEFENAMIC ACID TABLETS

Tablet	Claim mg/tablet	Amount found mg/tablet	Estimated label claim (average of 4 determinations)	Standard deviation
Meftal 500	500	501.5	100.30	0.14
Meftal spas	250	250.5	100.17	0.37
Meftal 250	250	250.4	100.15	0.30
Meftal P 100	100	100.4	100.35	0.16
Neopan	500	499.3	99.87	0.15
Meftal Forte	500	501.5	100.30	0.16

Meftal tablets is manufactured by Blue Cross Pharmaceuticals Ltd., Goa. Neopan tablets is manufactured by Noel Pharmaceuticals Ltd., Mumbai.

The assay of six different tablets containing mefenamic acid was within acceptable limits indicating that the procedure is accurate. All the tablets could be estimated with an error less than 0.5%. The inactive ingredients present in the tablets did not interfere with the analysis. Meftal Forte contains paracetamol as an additional active ingredient but did not interfere with the assay of mefenamic acid since the λ_max._for paracetamol is 289 nm.
